# Side-by-Side Comparison of Commonly Used Biomolecules That Differ in Size and Affinity on Tumor Uptake and Internalization

**DOI:** 10.1371/journal.pone.0124440

**Published:** 2015-04-22

**Authors:** Jeerapond Leelawattanachai, Keon-Woo Kwon, Praveesuda Michael, Richard Ting, Ju-Young Kim, Moonsoo M. Jin

**Affiliations:** 1 Department of Biomedical Engineering, Cornell University, Ithaca, NY 14853, United States of America; 2 Department of Radiology, Weill Cornell Medical College, New York, NY 10065, United States of America; 3 Department of Advanced Materials Engineering, Kangwon National University, Samcheok, South Korea; 4 National Nanotechnology Center, National Science and Technology Development Agency, Pathumthani 12120, Thailand; 5 Department of Biomedical Engineering, Dongguk University, Seoul 100–715, South Korea; National Cancer Institute, NIH, UNITED STATES

## Abstract

The ability to use a systemically injected agent to image tumor is influenced by tumor characteristics such as permeability and vascularity, and the size, shape, and affinity of the imaging agent. In this study, six different imaging biomolecules, with or without specificity to tumor, were examined for tumor uptake and internalization at the whole body, ex-vivo tissue, and cellular levels: antibodies, antibody fragments (Fab), serum albumin, and streptavidin. The time of peak tumor uptake was dependent solely on the size of molecules, suggesting that molecular size is the major factor that influences tumor uptake by its effect on systemic clearance and diffusion into tumor. Affinity to tumor antigen failed to augment tumor uptake of Fab above non-specific accumulation, which suggests that Fab fragments of typical monoclonal antibodies may fall below an affinity threshold for use as molecular imaging agents. Despite abundant localization into the tumor, albumin and streptavidin were not found on cell surface or inside cells. By comparing biomolecules differing in size and affinity, our study highlights that while pharmacokinetics are a dominant factor in tumor uptake for biomolecules, affinity to tumor antigen is required for tumor binding and internalization.

## Introduction

Whole body imaging using molecular agents is becoming an increasingly important tool to diagnose and stage cancers and to assess treatment response [1–5]. Molecular imaging is enabled by contrast agent-conjugated small molecules, antibodies, and other recombinant peptides or proteins that are more selective to tumor cells than to normal cells. Unlike the labeling of tumors *in vitro*, where affinity and specificity of imaging agents are dominant factors, *in vivo* detection of tumors is much more complex. In *in* vivo detection, molecular imaging agents are first exposed to the body’s physiology before they are localized to the tumor, where finally, molecular affinity and specificity can determine the interaction between agents and tumor [6–10]. Factors affecting the performance of cancer targeting agents include molecular size, shape, and interaction with other molecules in serum, and specificity and affinity for target antigens [11–14]. Properties related to the tumor such as size, tendency for non-specific uptake, vascular and lymphatic networks, and permeability within the tumor and across capillaries, also influence the performance of targeting agents in molecular imaging [7,12,15–17].

Previous studies using native antibodies (full-length immunoglobulin (Ig)), enzymatically truncated fragments (e.g., fragment antigen-binding (Fab), (Fab’)_2_), and various recombinant antibody variants (e.g., single chain fragment variable domains (scFv), multivalent formats of scFv) have shown that specificity and affinity to tumor antigens are not the only factors that determine their ability to detect tumors [18–20]. Theoretical and experimental studies have revealed that molecular size is one dominant factor that influences tumor targeting [21–23]. After emigration and localization in tumors that have less developed lymphatic drainage, molecules such as antibodies (~150 kDa) can be retained within the tumor for an extended period of time, an effect known as enhanced permeability and retention (EPR) [12,21,24]. This creates uncertainty of the relative contribution of affinity and EPR effect, complicating interpretation of the biodistribution of imaging agents. Previous studies have also predicted that molecular imaging agents sized 20–50 kDa are the least effective in tumor targeting because they are too large to rapidly extravasate and diffuse into tumors while simultaneously being too small to avoid fast renal clearance [21,24,25]. Furthermore, the fact that monovalent variants of antibodies (e.g., scFv (25 kDa) and Fab (50 kDa)) no longer possess high affinity due to the loss of avidity may place them to be least suitable for molecular imaging agents [19–21,26].

Drug carriers that preferentially deliver drugs to target sites may attain selectivity by means of molecular targeting, an EPR effect, or both. Beyond the issue of tumor localization, identifying characteristics that allow for rapid cellular internalization becomes a critical issue when agents are designed to target intracellular molecules or when they bare therapeutics, like antibody-drug conjugates. It has been well demonstrated that not all molecules found within tumors are able to bind and enter cells [27–31]. Despite a plethora of studies demonstrating the influence of size and affinity on tumor uptake and internalization, there have been few studies that have simultaneously examined commonly used biomolecules for their biodistribution, pharmacokinetics, tumor detection, and internalization in the same animal model. In the present study, mice xenografted with human cervical cancer cells (HeLa) that overexpress intercellular adhesion molecule (ICAM)-1 were used to evaluate molecular imaging agents. ICAM-1 is constitutively over-expressed in many carcinomas including breast, colon, non-small cell lung, and gastric tumors, and in tumor stroma within an inflammatory network [32,33]. To compare how size and affinity affect tumor detection in this tumor model, antibodies with or without affinity to ICAM-1 (in both Ig and Fab formats) and serum albumin and streptavidin, which are currently being used in clinical drug-carrier and imaging applications, were used as imaging agents. The present study shows that molecular size and favorable pharmacokinetics that are unrelated to tumor affinity can far outweigh biomolecular affinity to tumors. These qualities are demonstrated by superior tumor targeting by serum albumin and streptavidin over tumor-specific Fab fragments. However, for internalization of biologics into tumors, affinity to tumor antigen is essential.

## Materials and Methods

### Mammalian cell culture

HeLa and HEK 293 cells (ATCC) were cultured in Advanced Dulbecco’s modified Eagle’s medium containing 10% (v/v) fetal bovine serum, 2 mM L-alanyl-L-glutamine dipeptide, and 100 U/ml Penicillin-Streptomycin (Invitrogen) at 37°C in a 5% CO_2_ humidified incubator. A mouse monoclonal antibody R6.5 (IgG2a) [34] was produced from hybridoma (R6.5.D6.E9.B2; ATCC). The hybridoma cells were expanded in RPMI 1640 medium supplemented with 10% (v/v) fetal bovine serum, 2 mM L-alanyl-L-glutamine dipeptide, 100 U/ml Penicillin-Streptomycin at 37°C in 5% CO_2_ and were transferred for antibody production to CD Hybridoma media with 2 mM L-alanyl-L-glutamine dipeptide and 100 U/ml Penicillin-Streptomycin.

### Production of IgG and enzymatic fragments

R6.5 IgG in culture media were affinity purified from cell culture supernatants using Affi-Gel Protein A MAPS II Kit (Bio-Rad). Eluted fractions were immediately neutralized, concentrated using 10 kDa cut-off Amicon Ultra-15 Centrifugal Filter Units (EMD Millipore), and buffer exchanged into PBS using PD-10 Desalting Columns (GE Healthcare). Fab fragments of R6.5 and mouse serum IgG (Sigma-Aldrich) were generated by digesting IgG with papain [35]. One volume of 2 mg/ml IgG in PBS, pH 6.2, was incubated at 37°C for 15 h with one volume of 20 μg/ml papain (Sigma-Aldrich) in PBS containing 20 mM ethylenediaminetetraacetic acid disodium salt (Sigma-Aldrich) and 20 mM cysteine (Sigma-Aldrich), pH 6.2. Fab fragments were separated from Fc and undigested IgG by Affi-Gel Protein A MAPS II Kit, followed by buffer exchange into PBS using PD-10. The concentration of antibody and fragments was determined by measurement with a NanoDrop (Thermo Scientific). Antibody and digestion products were analyzed on SDS-PAGE.

### In vitro binding and internalization assays

Antibodies, Fab fragments, streptavidin (Invitrogen), and bovine serum albumin (BSA, Fisher Scientific) were covalently labeled with Alexa Fluor 488 sulfodichlorophenol ester (Alexa488) and Alexa Fluor 750 succinimidyl esters (Alexa750) (Life Technologies). Degree of labeling for each dye was determined by the absorbance values of proteins and dyes, according to manufacturer’s protocol. To test binding of imaging agents *in vitro*, HeLa and HEK 293 cells were trypsinized, washed with ice-chilled complete cell culture medium, and incubated in 50 μl culture media on ice for 1 h with 10 μg/ml labeled molecules (200,000 cells per labeling sample). Cells were washed twice in 300 μl of culture media, and suspended in 300 μl PBS for flow cytometry (BD-Biosciences LSRII Flow Cytometer). For quantitative measurement of binding affinity (equilibrium dissociation constant, Kd), R6.5 specific quantities of IgG and Fab fragments labeled with Alexa488 (0–100 nM) were added to HeLa cells. After incubation at 4°C for 60 min, cells were centrifuged, suspended in PBS, and were immediately analyzed by flow cytometry. The Langmuir isotherm model was fit to the values of mean fluorescence intensity versus antibody concentration using GraphPad Prism (GraphPad Software, Inc) to estimate Kd and confidence intervals.

For internalization of imaging agents *in vitro*, cells were first labeled with imaging agents at 4°C as described above. Subsequently, cells were incubated in culture media at 37°C for 0, 1, or 3 hours to trigger internalization of imaging agents. After incubation, cells were divided into two tubes and rapidly chilled on ice. Cells in one tube were incubated on ice for 2 h with 25 μg/ml rabbit anti-Alexa488 (Life Technologies) to quench fluorescence of the surface bound imaging agents. Cells in both tubes were analyzed by flow cytometry. Surface bound percentages of imaging agents were calculated as %surface = (reduction in fluorescence due to quenching after 37°C incubation)/(reduction in fluorescence due to quenching at 4°C). Then, the percentages of imaging agents internalized were calculated as %internalized = 100%—%surface [36,37].

### Subcutaneous tumor model and injection of imaging agents

All animal experiments were performed in strict accordance with the recommendations in the Guide for the Care and Use of Laboratory Animals of the National Institutes of Health. The protocol of this study was approved by the Institutional Laboratory Animal Use and Care Committee of Cornell University (Permit Number: 2008–0079). HeLa cells (3×10^6^) suspended in 1:1 PBS:Matrigel (BD) mixture in a total volume of 150 μl were injected subcutaneously on the posterior, lower flank area of 4 week old female, severe combined immunodeficiency (SCID) hairless mice (strain code: 474, Charles River Laboratories). Tumor growths were measured using a vernier caliper, and the tumor volumes were calculated by formula: 0.5 × length × (width)^2^. When tumors reached approximately 150 mm^3^, mice were randomized into eight different groups (six groups with each of six different imaging agent for 24 h follow-up and two groups with R6.5 and control antibodies for 7 day follow-up; n = 3–6) and injected via retro-orbital with fluorescently-labeled imaging agents (100 μg in 200 μL PBS) using 29G insulin syringes. Mice with subcutaneous tumors larger than 15 mm diameter (or larger than 2,000 mm^3^) were immediately euthanized by CO_2_ asphyxiation.

### Pharmacokinetic (PK) studies

The pharmacokinetic parameters were determined by fitting a two compartmental model to the percentage of injected dose per milliliter of blood (%ID/mL) versus time (h) using PK Solver Microsoft Excel add-in as previously described [38]. The two compartmental model is characterized by an initial rapid clearance of injected molecules from the central compartment (blood and well perfused organs) to the peripheral compartment (tissue), described by rate constants *K*_12_ and *K*_21_, and elimination from the body due to clearance from the central compartment, described by rate constant *K*_10_. From this model were obtained the standard pharmacokinetic parameters including distribution half-life (t_1/2(α)_), elimination half-life (t_1/2(β)_), mean residence time, clearance from the central compartment, and volume distribution. After administration of imaging agents, approximately 30 μL blood was collected from the submandibular vein at different time points. Plasma was separated from the whole blood by centrifugation (5000×g, 10 min) and stored at -20°C until analysis. The concentration of imaging agents in the plasma was estimated by fluorescence measured with a microplate reader (Bio-Tek). A standard curve for the imaging agents was created by adding known amounts to the plasma of untreated mice.

### In vivo imaging and biodistribution studies

Whole body imaging was performed using a Xenogen IVIS-200 (Perkin Elmer). Mice were anesthetized first with 5% isoflurane (VetOne) and maintained at 2% isoflurane during imaging session. Whole body fluorescence images were acquired at 1, 4, 8, 12, and 24 hours postinjection of imaging agents. Selected mice injected with IgGs were imaged at 1, 4, 8, 12, 24, and every 24 hours afterward up to 168 hours postinjection. After the last time point of imaging (24 or 168 hours), mice were euthanized, and the major organs (lungs, heart, spleen, kidneys, liver, and brain) and tumors were harvested. The level of tumor uptake of imaging agents in live mice (%ID/g) was then calculated as %ID/g = (measurement of relative fluorescence in comparison to total fluorescence injected)/(tumor weight at time of harvest). Subsequently, biodistribution (%ID/g) of imaging agents into the major organs and tumors was calculated as %ID/g = (measurement of *ex vivo* relative fluorescence in comparison to total fluorescence injected)/(tissue weight). To correct for tissue autofluorescence, the background fluorescence of non-treated tissues was subtracted from *in vivo* and *ex vivo* measurements of fluorescence of treated tissues.

### In vivo internalization assay

To measure the amount of imaging agents that bound to cell surface or internalized, singlet cells were obtained by first mincing collected tumors and digesting with 1 μg/ml collagenase A (Roche) in culture media for 2 h at 37°C [39]. Isolated cells were passed through 70 μm nylon mesh cell strainer (BD Falcon) and incubated with red blood cell lysis buffer (eBiosciences) for 20 min at room temperature. Cells were then processed to quantify the fraction of internalized agent as described above in the internalization assay. From the mixture of cells isolated from the tumor, flow cytometry gating on forward and side scatter plots was defined to include live HeLa cells that were contaminated with minimal quantities of dead cells and other cell types such as macrophages and endothelial cells [40].

### Statistical analysis

Data were expressed as mean ± standard error of the mean (SEM). Statistical analysis was performed using GraphPad Prism. Differences with p values < 0.05 were considered significant. Student's t-test was used to evaluate the difference between mean values to different treatments. Two-way analysis of variance (ANOVA) was used to compare the mean responses of different treatments to different time points or to different tissues, followed by Bonferroni post-hoc test to determine statistical significance.

## Results

### Preparation of biomolecules, *in vitro* assays to confirm ICAM-1 dependent binding

**Fig 1:**
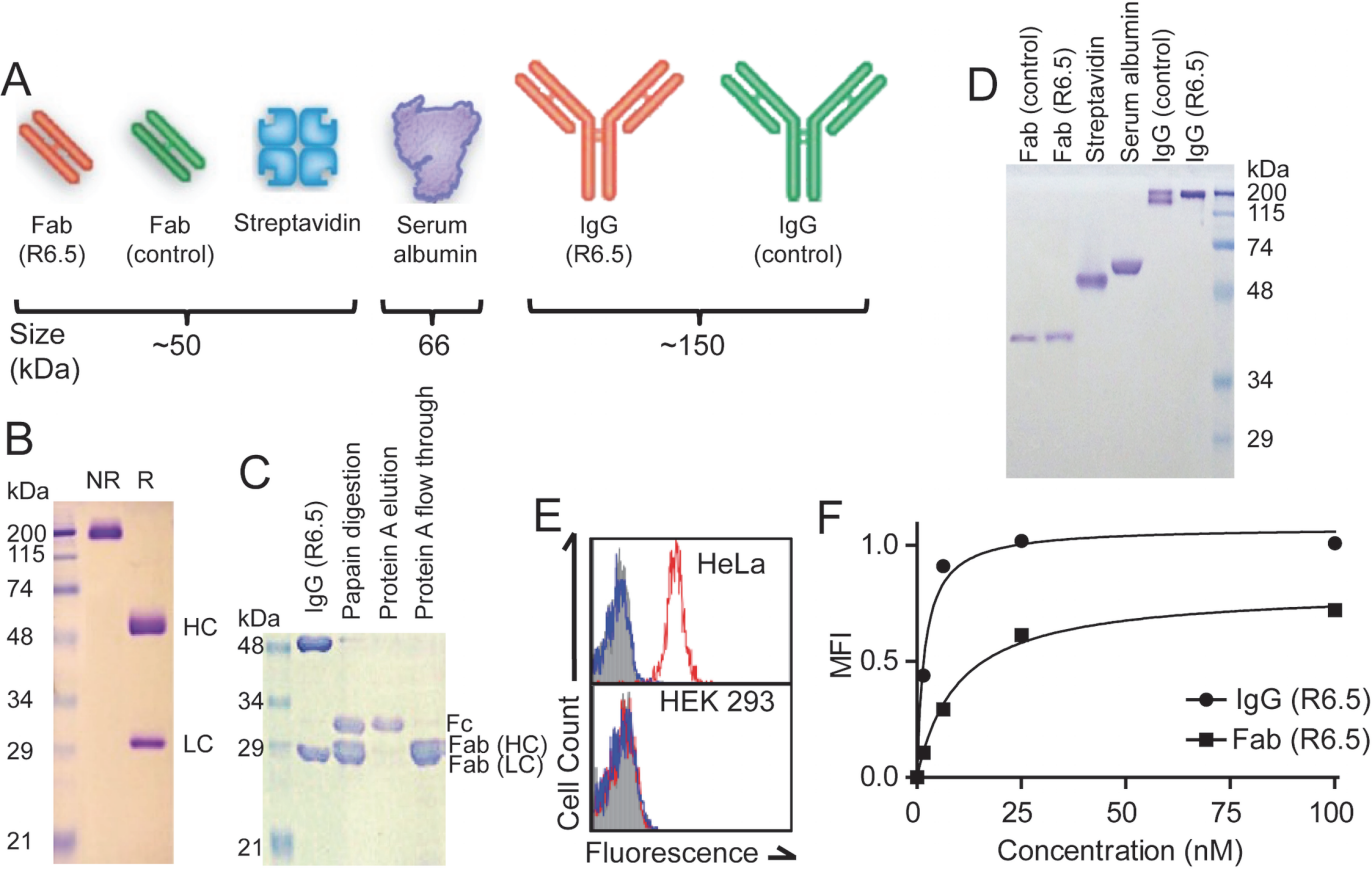
Preparation of biomolecules used as imaging agents for tumor detection. (A) Schematic drawing of antibody and antibody fragments (Fab), and serum albumin and streptavidin, illustrating size and shape of molecules. Approximate molecular weights are noted. (B-D) Coomassie blue stained 12% SDS-PAGE of protein A purified R6.5 IgG under non-reducing (NR) and reducing (R) conditions (B), digestion of R6.5 with papain, protein A elution, protein A flow products under reducing condition (C), all six biomolecules used in this study under non-reducing condition (D). The bands corresponding to the heavy and light chains of IgG (HC, LC) and, Fc, and Fab were labeled. (E) Flow cytometry histograms using constitutive ICAM-1 expressing HeLa cells and ICAM-1 negative HEK 293 cells labeled with Alexa488-labeled R6.5 (histograms in red line) and control IgG (histogram in blue line). Autofluorescence of cells without labeling is shown in solid filled histograms. (F) The level of binding (mean fluorescence intensity (MFI)) to HeLa cells of Alexa488-labeled R6.5 IgG and Fab was plotted against the concentration to estimate binding affinity. The solid line is the curve-fit of the Langmuir binding isotherm equation to data.

Natural IgG and enzymatically truncated or recombinant variants of IgG have been a dominant source of biomolecules for molecular imaging applications. Besides antibodies, other commonly used imaging agents include serum albumin and avidin/streptavidin [16,41,42] for their superior *in vivo* safety and pharmacokinetics. In this study, ICAM-1 specific monoclonal antibody (mAb), R6.5 (mouse IgG2) in native and Fab format were used to selectively bind ICAM-1 positive HeLa cells. As a control for R6.5, polyclonal IgG purified from mouse serum was used. In total, six different molecules were used. These molecules differ in molecular weight (from 50 to 150 kDa), and have varying degrees of affinity to ICAM-1 (Fig 1). mAb R6.5 was produced from hybridoma (~20 mg/L of culture supernatant) and prepared to >95% purity (Fig 1B). Fab of both R6.5 and control IgG were produced by papain digestion followed by the removal of antibody Fc fragment with protein A column. Under reducing gel electrophoresis (SDS-PAGE), Fab migrated as two bands of heavy and light chain fragments (Fig 1C; labeled as Fab (HC) and Fab (LC), respectively). To confirm size and purity, all six molecules were analyzed side-by-side on non-reducing SDS-PAGE (Fig 1D). Mouse IgG migrated into at least two distinct bands, indicative of polyclonality and mixed immunoglobulin isotypes. Streptavidin (~52 kDa) maintains a tetrameric structure, and both streptavidin and BSA (66 kDa) migrated close to their theoretical molecular weights. Fab fragments of R6.5 and control antibody exhibit faster mobility, migrating with nominal molecular weight of ~40 kDa, often observed with molecules containing disulfide bonds. To confirm specificity to ICAM-1 *in vitro* and *in vivo* assays, all the molecules were labeled with two different fluorescent dyes (Alexa488 and Alexa750). Labeling conditions were adjusted to add, on average, 1.2–1.5 of each dye per molecule. The specificity of R6.5 against ICAM-1 was confirmed by observing selective binding to ICAM-1 positive HeLa and a lack of binding to ICAM-1 negative HEK 293 cells (Fig 1E). Control IgG, BSA, and streptavidin exhibited no discernible binding above background levels to either HeLa or 293 cells (Fig 1E and data not shown). Finally, to measure an affinity difference between R6.5 IgG and Fab, HeLa cells were labeled with Alexa488-labeled molecules used at 0–100 nM and the level of binding was measured by flow cytometry (Fig 1F). The affinity (equilibrium dissociation constant, Kd) of R6.5 IgG and Fab to ICAM-1 was estimated at 1.9 ± 0.4 and 9.9 ±1.4 nM Kd, respectively.

### Measurement of pharmacokinetics of biomolecules

**Fig 2:**
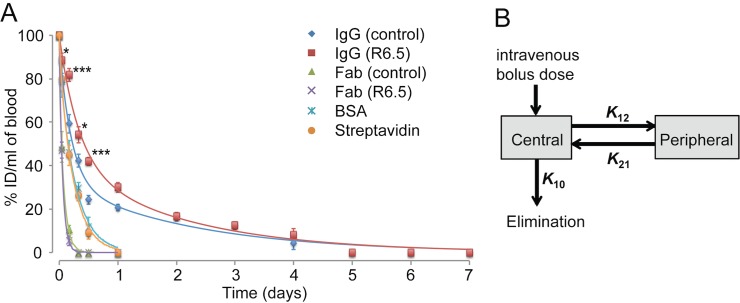
Measurement of imaging agent concentration in blood to estimate pharmacokinetic parameters. (A) The concentration of each imaging agents in blood was measured by fluorescence, and plotted as a percentage of injected dose (%ID/mL) versus time (n = 3 for all except n = 6 for R6.5 and control IgG at 1, 4, 12 and 24 h postinjection with standard error of mean (SEM) as error bars). Two-way ANOVA followed by Bonferroni post-hoc test was used to evaluate statistical significance of responses of different imaging agents at different time points (S1 Table). Significant differences are indicated with *p < 0.05 and ***p < 0.001 for R6.5 versus control IgGs. (B) Schematic diagram describing a two-compartment pharmacokinetic model with rate constants is shown. Central compartment represents blood and well perfused organs (e.g., plasma), while peripheral represents poorly perfused tissues. *K_10_*, *K_12_*, and *K_21_* represent, respectively, rate constants for elimination, distribution, and redistribution.

Prior to studying the biodistribution of biomolecules *in vivo*, the pharmacokinetic parameters of each molecule were determined by measuring the concentration in the blood over 1–7 days postinjection. The decrease in the concentration of imaging agents in the blood was fastest with Fab, followed by streptavidin and BSA, and slowest with IgGs (Fig 2A). Possessing affinity to ICAM-1 did not result in difference for R6.5 and control Fab, yet significant difference was found between R6.5 and control IgGs at time points up to 12 hours (p<0.001–0.05; S1 Table). However, the elimination half-lives (t_1/2β_) of the molecules, calculated from the curve-fit of the two compartmental PK model to the data (Fig 2B), were directly related to the molecular weights, insensitive to possessing affinity to tumor antigens (Table 1). Conversion of IgG into Fab, a reduction of size by 3-fold, resulted in ~10-fold faster elimination rates. Despite a small difference in molecular mass, Fab fragments were cleared faster from the blood (~1 h) than BSA and streptavidin (4–5 h). The affinity to tumor antigen did not alter the rate of clearance and mean residence time of R6.5 IgG from control IgG, despite being significantly different in the blood at earlier time points, and R6.5 Fab from control Fab.

**Table 1:** Pharmacokinetic parameters of the imaging agents derived from a two-compartmental model.

Biologics	t_1/2 (α)_	t_1/2 (β)_	AUC_0-t_ *	AUC_0-inf_	MRT	CL	V_d_	*K*_10_	*K*_12_	*K*_21_
	(h)	(h)	(h×μg/ml)	(h×μg/ml)	(h)	(ml/h)	(ml)	(1/h)	(1/h)	(1/h)
IgG (control)	3.8	51.5	1233.7	1607.2	63.7	0.1	1.6	0.04	0.10	0.06
IgG (R6.5)	6.5	52.1	1547.5	1981.3	61.0	0.1	1.5	0.03	0.04	0.04
Fab (control)	0.1	1.3	106.4	106.6	1.9	0.9	1.5	0.63	0.91	5.16
Fab (R6.5)	0.6	1.0	90.0	90.0	1.4	1.1	1.5	0.74	0.02	1.12
BSA	0.7	4.7	327.6	393.7	6.7	0.3	1.5	0.17	0.11	0.88
Streptavidin	0.7	4.1	315.1	362.7	5.9	0.3	1.5	0.18	0.07	0.86

*t = 168 h for IgG, t = 24 h for Fab, BSA and Streptavidin. Notations and abbreviations: distribution half-life (t_1/2α_), elimination half-life (t_1/2β_), area under the curve (AUC), mean residence time (MRT), clearance (CL), volume of distribution (V_d_), elimination rate constant (*K*_10_), distribution rate constant (*K*_12_), redistribution rate constant (*K*_21_).

### Whole body imaging of biomolecules in mice with human tumors

**Fig 3:**
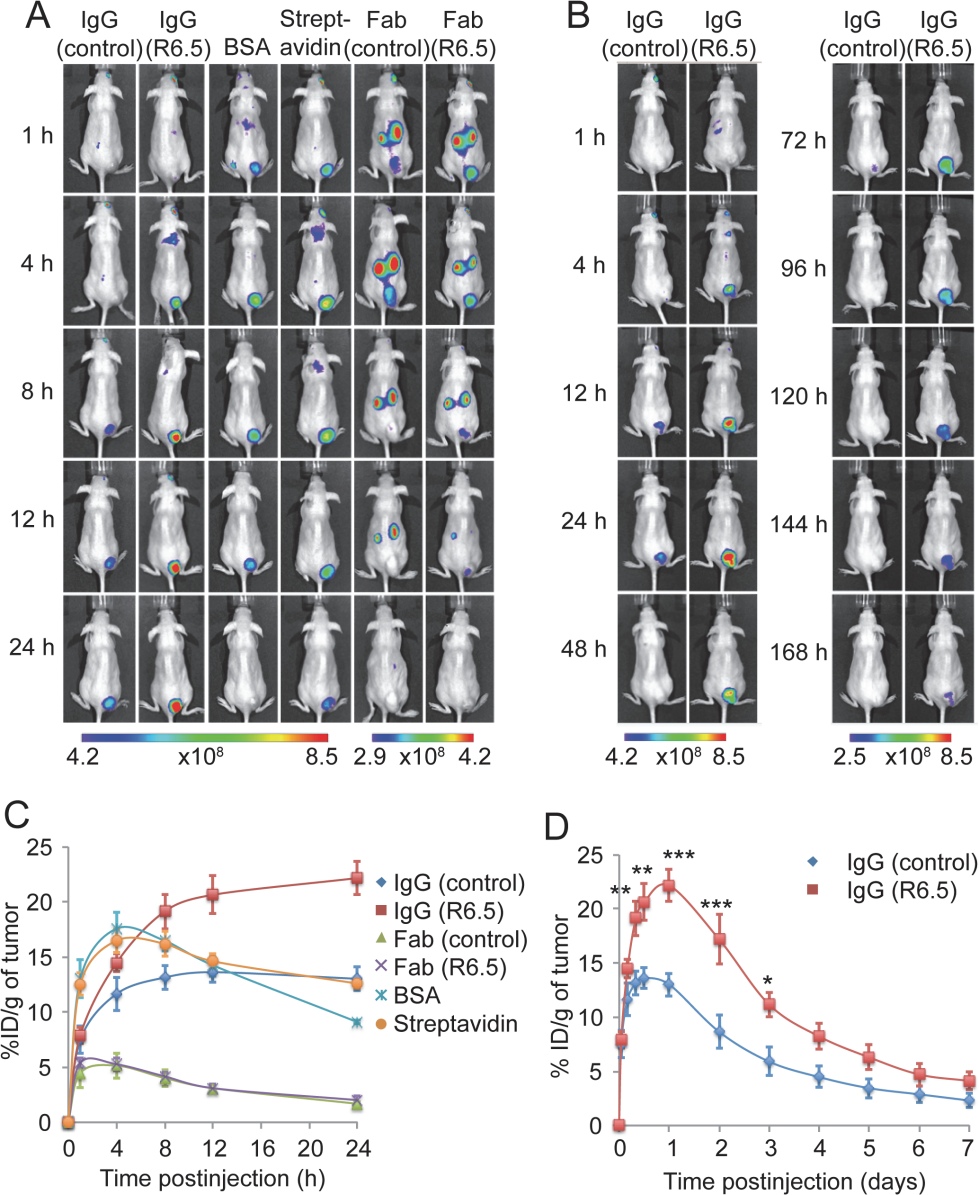
Real-time whole body imaging of biomolecules in mice xenografted with HeLa tumors. (A, B) Representative time-course images of merged fluorescence and bright fields are shown for HeLa-implanted SCID mice after intravenous administration of the fluorescence labeled imaging agents. Note that different color scales, representing fluorescence intensity in arbitrary units, are used for optimal signal-to-noise ratios. (C, D) Quantification of tumor uptake of each imaging agents was obtained with Living Image software. Error bars are SEMs. Two-way ANOVA followed by Bonferroni post-hoc test was used to evaluate statistical significance of responses of different imaging agents at different time points (S2 Table). Significant differences are indicated with *p < 0.05, **p < 0.01, ***p < 0.001 for R6.5 and control IgG (n = 3 for all except n = 6 for R6.5 and control IgGs at 1, 4, 12 and 24 h postinjection).

**Fig 4:**
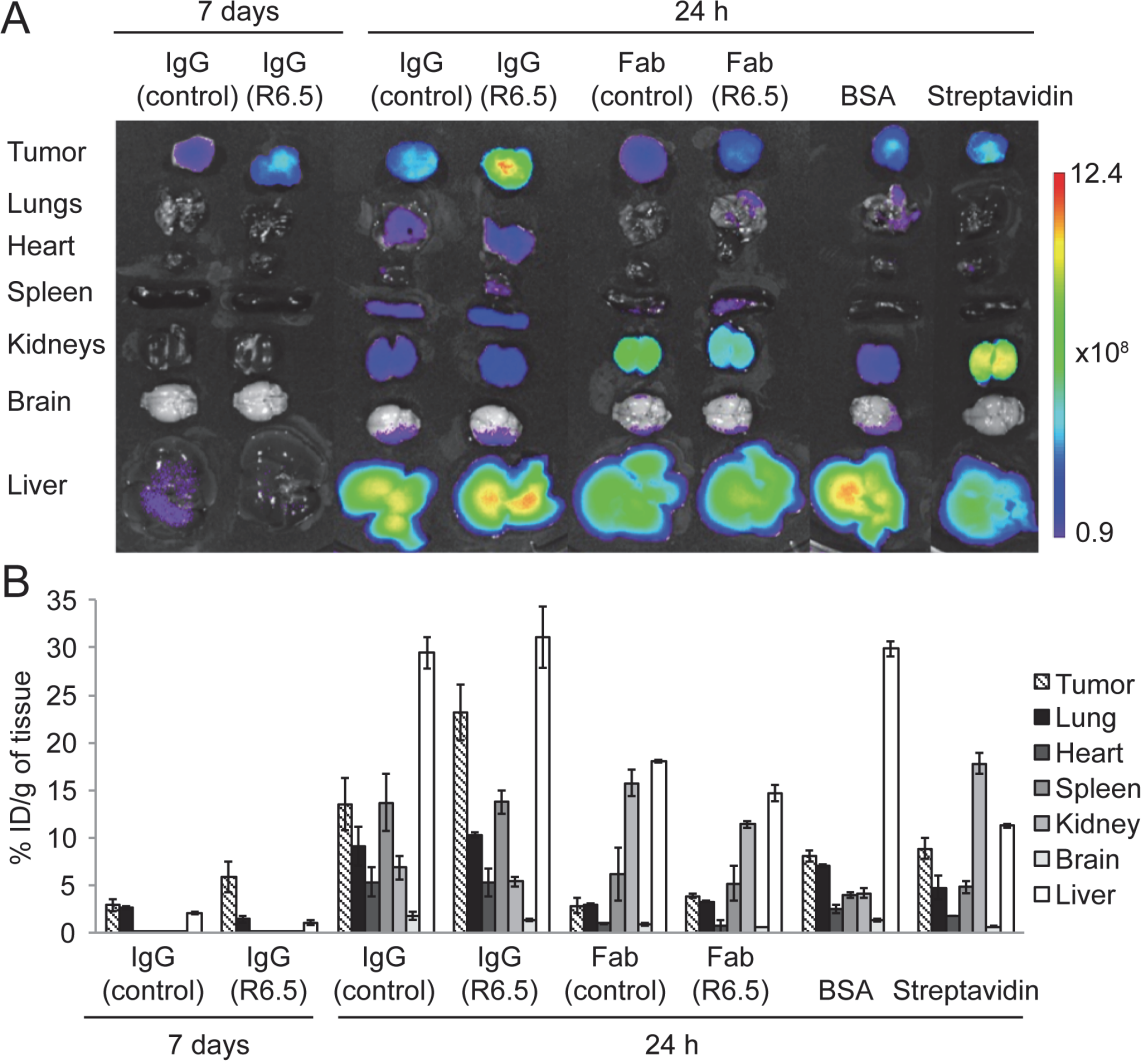
*Ex vivo* imaging of tumor imaging agents in major organs and tumors after 24 hours or 7 days. (A) Representative images of merged fluorescence and bright fields for *ex vivo* organs and tumor. (B) Quantification of biodistribution into the major organs and tumors (%ID/g mean ± SEM) is shown as a bar graph (n = 3 each). Two-way ANOVA followed by Bonferroni post-hoc test was used to evaluate statistical significance of responses of different imaging agents at different tissues (S3 Table).

Six different molecules labeled with near-infrared fluorescent dyes were compared for their biodistribution and tumor uptake in live mice xenografted with HeLa tumors (Fig 3). For quantification of tumor uptake of imaging agents in live mice (%ID/g), the background-corrected signal intensity over the tumor area was divided by the weight of tumors measured at 24 h (Fig 3C) or 7 days (Fig 3D) postinjection. The kinetics of tumor uptake and clearance of all six molecules, overall, followed the rate of clearance from the blood (shown in Fig 2A): the time of peak tumor uptake was estimated to be 1–2 h for Fab fragments, ~4 h for BSA and streptavidin, and ~24 h for control and R6.5 IgG (Fig 3). Consistent with faster clearance of Fab from circulation, intense signals of Fab were detected in the kidneys at the earliest time point examined (1 h). Despite difference in affinity to tumor, tumor uptake of R6.5 Fab was not significantly different from that of control Fab in delineating tumors (S2 Table). In comparison, BSA and streptavidin provided three times higher peak tumor uptake than Fab fragments (p<0.001). However, the highest accumulation into tumors was associated with ICAM-1 specific R6.5 IgG, which persisted up to 7 days after injection. Significant difference was observed for R6.5 and control IgG for the measurements up to 3 days postinjection (p<0.001–0.05; Fig 3C and 3D and S2 Table). Due to the proximity of the tumors to the skin, fluorescence measurements of tumor uptake of imaging agents taken in live mice at final time-points (Fig 3) were close to those taken *ex vivo* with less than 25% deviation from each other (Fig 4), validating quantitative nature and accuracy of the time-course measurements throughout.

### Organ-level assessment of biodistribution of imaging agents

Whole body imaging of fluorescent dye is biased to detection of imaging agents distributed closer to the skin. Quantitative mapping of imaging agents in the body requires analysis using *ex vivo* organs and tumors. After mice were euthanized, major organs (lungs, heart, spleen, kidneys, brain, and liver) and tumors were isolated and subjected to *ex vivo* imaging (Fig 4A). The uptake of imaging agents by the immune cells in the mononuclear phagocytic system would produce signals in the liver, lungs, and spleen. The uptake by the liver at 24 h was highest of all imaging agents with the exception of streptavidin, while signals in the kidneys were lower with larger molecules. In agreement with whole body imaging at 24 h postinjection, tumor uptake of R6.5 was significantly higher (p<0.001; S3 Table), which was followed by uptakes of control IgG, streptavidin, and BSA, and was lowest with Fab. The uptake into the spleen was significantly higher for control and R6.5 IgG over other smaller molecules (p<0.001). At 7 days postinjection, tumor uptake of R6.5 was still apparent, far higher than in the lungs and liver (Fig 4). Uptake into the tumor was different between control and R6.5 IgG (p<0.01), while no difference between them was found for uptakes into the other organs.

### *In vitro* assay to measure internalization of biomolecules into cells

**Fig 5:**
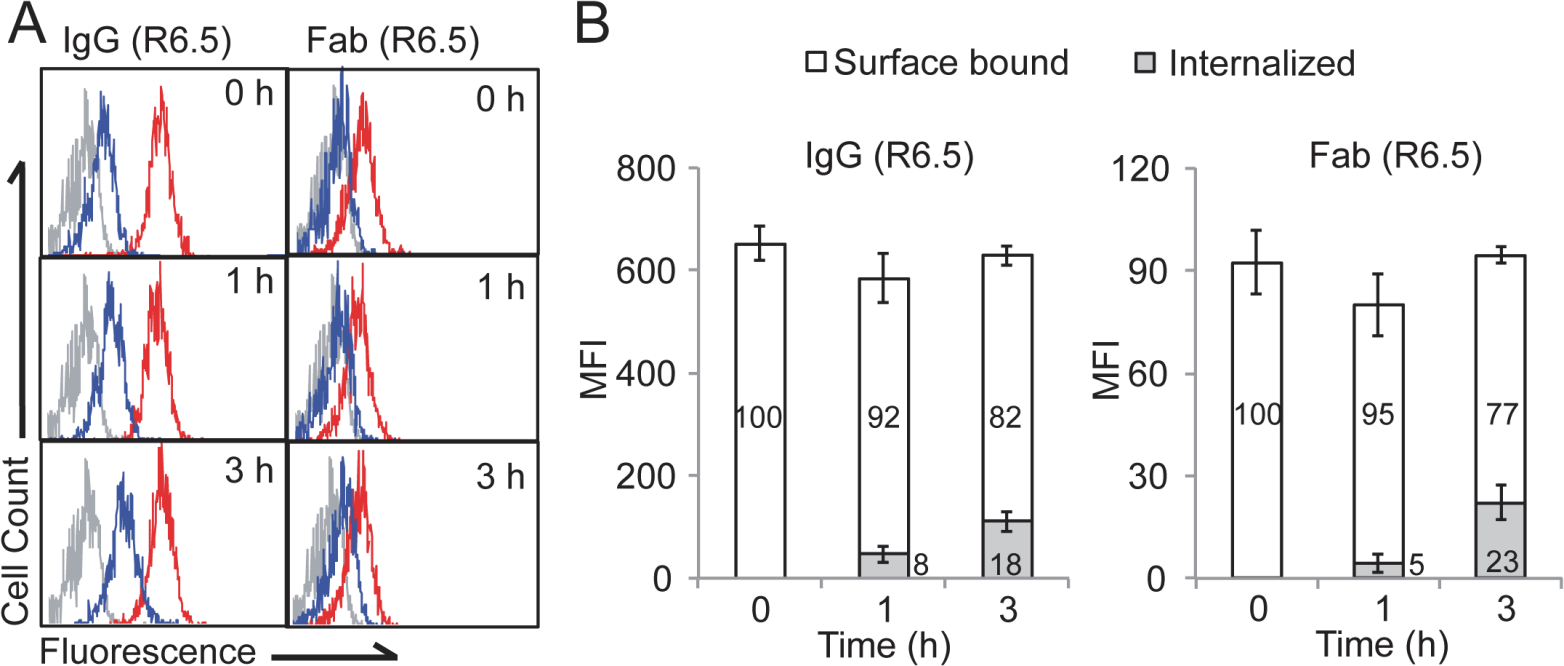
*In vitro* assays to confirm ICAM-1 specific internalization. (A) Flow cytometry histograms are shown for HeLa cells labeled with Alexa488-conjugated R6.5 IgG or Fab with (histograms in blue line) or without (histograms in red line) additional incubation with anti-Alexa488 antibody. 0, 1, 3 h indicate the time of incubation at 37°C after labeling with Alexa488-conjugated R6.5 IgG or Fab. Background fluorescence is shown in a histogram in gray. (B) Quantification of fractions internalized and surface bound is shown for R6.5 IgG and Fab in bar graphs as mean ± SEM (n = 3). The numbers represent the percentage values.

On a macroscopic imaging scale, the visualized accumulation of an imaging agent is the sum of its distribution on the cell surface, inside cells, and/or in the interstitial space within tumors. To evaluate the potential use of tumor targeting agents as drug carriers, an internalization assay was performed to determine the fraction of imaging agent that is cell surface bound vs. internalized. For this assay, HeLa cells were labeled with R6.5 IgG and Fab at 4°C, incubated at 37°C for 0–3 hours to promote antibody internalization, and subsequently labeled with anti-Alexa488 antibody to quench fluorescence from surface-bound biomolecules (Fig 5A). When cells were labeled with anti-Alexa488 antibody immediately after labeling with R6.5 at 4°C to prevent internalization, substantial reduction of fluorescence was observed for both IgG and Fab (92±4% and 95±3% for IgG and Fab, respectively). With increasing time of incubation at 37°C, the amount of internalized fractions gradually increased, reaching to 18% and 23% for R6.5 IgG and R6.5 Fab, respectively after 3 h incubation (Fig 5A and 5B).

### Specific molecular interaction is required for cell binding and internalization

**Fig 6:**
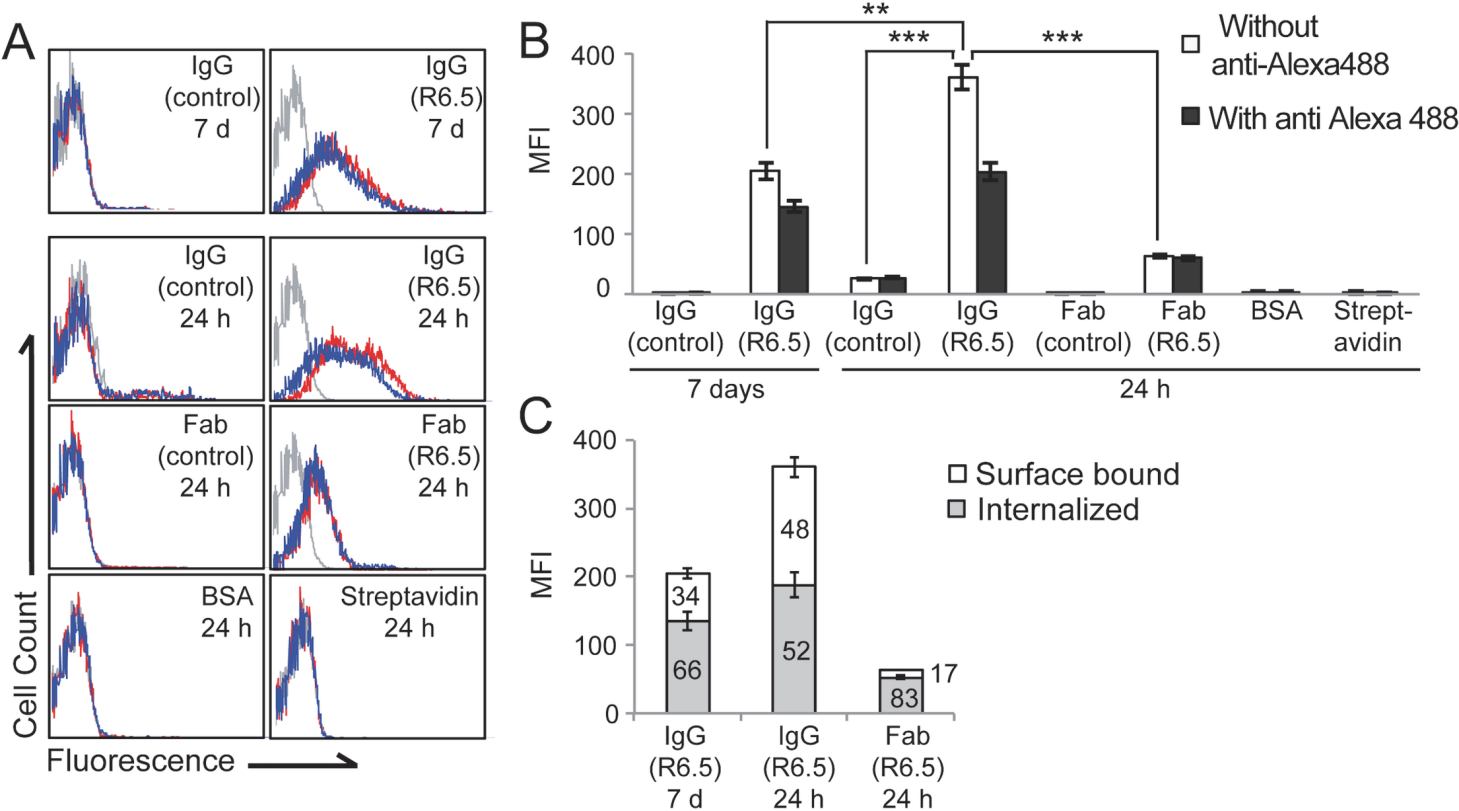
Specific interaction is required for cell binding and internalization. (A) Singlet cells from HeLa tumor were analyzed by flow cytometry with (histograms in blue line) or without (histograms in red line) incubation with anti-Alexa488 antibodies. Background fluorescence is shown in gray histogram. The top two histograms are for the tumors analyzed after 7 days postinjection of imaging agents, while the remaining six are for the tumors analyzed after 24 h postinjection of imaging agents. (B-C) Quantification of mean fluorescence intensity and fractions of surface bound versus internalized imaging agents are shown in bar graphs as mean ± SEM (n = 3; **p < 0.01, ***p < 0.001). The numbers represent the percentage values.

Imaging on the whole body, in vivo level does not provide enough information to show whether an agent is associated with an antigen on a cell or it is merely retained in interstitial spaces. To examine the influence of affinity to tumor antigens on tumor binding, flow cytometry and internalization assay were performed using singlet cells isolated from the tumors (Fig 6). Despite abundant signals of BSA and streptavidin in tumors, no fluorescence was associated with isolated singlet cells, indicating that they were mainly residing in interstitial space in tumors. In control IgG, a small fraction of cells (<5%) were labeled at 24 h postinjection, ascribed to low level non-specific binding or due to binding of IgG to Fc receptors expressed in mouse cells (e.g., endothelial cells). At 7 days of IgG injection, the fraction of cells associated with fluorescence was insignificant (<1%). In contrast, the fraction of tumor cells labeled with R6.5 IgG was highest reaching 75% at 24 h and continued to be present up to 60% at 7 days. In R6.5 Fab, which showed little difference from control Fab at the whole body level, as much as 45% of singlet cells were fluorescent.

From the degree of fluorescence quenching by anti-Alexa488 antibody, the percentage of R6.5 IgG internalized into cells at 24 h was close to 50% (Fig 6B and 6C). Although the level of fluorescence was lower at 7 days, the fraction of tumor cells that have internalized R6.5 was higher at 7 days (66%). Significantly higher percentage of tumor cells bound to R6.5 Fab was found to contain the molecules inside cells (83%), which is likely due to the clearance of Fab fragments from the tumor over 24 h that have not been internalized into cells.

## Discussion

The *in vivo* detection of tumors using molecular contrast agents is a complex problem, influenced by pharmacokinetics, the lymphatic and vascular networks within and surrounding tumors, and the characteristics of the imaging agents. Using commonly used biomolecules for tumor imaging and drug delivery, we examined the influence of molecular weight and tumor affinity on tumor uptake and internalization. The highest tumor uptake was found with tumor-specific, native antibody, but the observation of superior tumor uptake of serum albumin and streptavidin vs. similarly sized, tumor-specific Fab show that there is a point where pharmacokinetics and EPR can be more dominant factors in tumor imaging than tumor antigen affinity. This illustrates the difficulty with molecular imaging, particularly predicting the relative contribution of affinity to tumor antigens and of EPR effects. With proper controls, however, the level of background tumor uptake attained by EPR effect can be quantifiable in comparison to tumor uptake due to both effects of EPR and affinity to tumors. Only when the level of tumor uptake enhanced by affinity is significantly higher than by EPR alone will molecular imaging agents provide a reliable diagnosis of tumor phenotypes.

Modeling studies by others have demonstrated higher threshold of affinity for smaller imaging agents to augment tumor uptake above non-specific accumulation [21,43]. Our data also illustrated that the use of truncated, monovalent antibodies with molecular weights of 20–50 kDa may be more difficult to employ as imaging agents than previously thought. While threshold affinity is not universal for all tumors, our data suggested that such threshold of affinity may not be obtained with typical Fab fragments derived from monoclonal antibodies produced in mice. Given the measured affinity of R6.5 Fab to ICAM-1 (Kd ~10 nM), which produced little difference in tumor uptake from control Fab, we predict that Fab molecules need to possess binding affinity significantly higher than 10 nM to be used as molecular imaging agents. Therefore, molecules such as serum albumin and streptavidin, which balance systemic clearance and diffusion into tumors for maximal EPR effect, may be advantageous if the goal of an experiment is simply tumor detection. Further investigation is necessary to better define the influence that the molecular size of an agent and differences in vascular and lymphatic networks within different tumors have on the rates of extravasation and diffusion of imaging agents into tumors and ultimately molecules’ accumulation into the tumor. However, similar magnitudes and kinetics of tumor uptake of full-length and other truncated antibodies indicate that the parameters influencing tumor uptake, *i*.*e*., vascular permeability and diffusivity within tumor, are somewhat conserved among many different tumor models.

The best time window for whole body detection of tumor targeting agents needs to be determined to maximize sensitivity and signal-to-noise ratios. Our study suggested that the time of peak tumor uptake of Fab fragments and other biomolecules in tumor detection was proportionate to their elimination half-lives in the blood, including ~1 h for Fab fragments and 4–5 h for serum albumin and streptavidin. For IgGs, the peak uptake was observed at ~24 h, while the elimination half-lives were longer at ~50 h. However, the concentration of IgGs at 24 h post-injection in the blood was higher than in tumor, and it was not until >48 hours that signals in the tumor were higher than in blood. Optical imaging was used to visualize imaging agents that were localized close to the skin surface. With other imaging modalities that are not limited to tissue depth, such as PET and SPECT, the optimal time window for imaging would also be around the elimination half-lives of antibodies. This is corroborated by *ex vivo* imaging of the major organs and tumors that had higher signals in the liver than in tumors at 24 h, while at 7 days, the tumor retained highest signals.

A small change in molecular weight led to substantial differences in pharmacokinetics and systemic clearance, illustrated by the superior, yet delayed tumor uptake of streptavidin and albumin compared to Fab. Streptavidin and serum albumin had almost identical temporal kinetics of tumor uptake, yet serum albumin is ~30% larger in molecular weight. Modeling studies on tumor uptake of imaging agents approximates molecules being globular, relating molecular weight to hydrodynamic radius [44]. Obviously, this approximation is not valid for elongated molecules such as Fab, evidenced by significant difference of Fab in pharmacokinetics and temporal change in biodistribution from streptavidin. Therefore, empirically measured hydrodynamic radius (estimated from sedimentation and gel filtration experiments [45]) will provide a better prediction of the molecules’ biodistribution and tumor uptake, which would discriminate molecules of comparable molecular weights but differing in shape.

In conclusion, by simultaneously comparing widely used biomolecules that differ in size and affinity, our study suggests that relating tumor uptake of imaging agents to molecular imaging, including evaluation of tumor antigen expression, is challenging and critically dependent on relative contribution of EPR effect to affinity. The notion that possessing affinity to tumor antigens should augment tumor uptake of imaging agents needs to be carefully examined, with the understanding that the threshold affinity to make differences in tumor uptake would be higher for smaller molecules. One can take a protein engineering approach to generate variants with differing affinities to tumor antigens and use them to more accurately define the dependence of molecular size on the threshold affinity and ultimately the threshold tumor uptake to discern molecular targeting from a simple EPR effect. We also demonstrate that the efficiency of molecules such as serum albumin in delineating tumors owing to its ideal molecular size to maximize EPR effect. Our study also recapitulates the notion that specific molecular interaction is necessary for imaging agents to bind and enter cells. Biomolecules taken up by cells would largely avoid gradual clearance by the body, and increase signal-to-noise ratios in tumor detection. Of particular importance is the ability to go inside target cells of the agents to function as a carrier of therapeutics such as antibody-drug conjugates.

## Supporting Information

S1 Table Two-way ANOVA of the data shown in Fig 2A. Two-way ANOVA was used to compare the %ID/ml in the blood of different imaging agents to different time points, followed by Bonferroni post-hoc test. ns = not significant, *p < 0.05, and ***p < 0.001, n = 3 for all except n = 6 for R6.5 and control IgG at 1, 4, 12 and 24 h postinjection. Abbreviations and notations used: mIgG = control IgG; RIgG = R6.5 IgG; BSA = bovine serum albumin; Strep = streptavidin; mFab = control Fab; RFab = R6.5 Fab.
(DOCX)

S2 Table Two-way ANOVA of the data shown in Fig 3C&3D. Two-way ANOVA was used to compare the %ID/g in the tumor of different imaging agents to different time points, followed by Bonferroni post-hoc test. ns = not significant, *p < 0.05, **p < 0.01 and ***p < 0.001, n = 3 for all except n = 6 for R6.5 and control IgG at 1, 4, 12 and 24 h postinjection. Abbreviations and notations used: mIgG = control IgG; RIgG = R6.5 IgG; BSA = bovine serum albumin; Strep = streptavidin; mFab = control Fab; RFab = R6.5 Fab.
(DOCX)

S3 Table Two-way ANOVA of the data shown in Fig 4B. Two-way ANOVA was used to compare the %ID/g of different imaging agents to different tissues, followed by Bonferroni post-hoc test. ns = not significant, *p < 0.05, **p < 0.01 and ***p < 0.001, n = 3. Abbreviations and notations used: mIgG = control IgG; RIgG = R6.5 IgG; BSA = bovine serum albumin; Strep = streptavidin; mFab = control Fab; RFab = R6.5 Fab.
(DOCX)
