# Supplementary material for: Side-by-Side Comparison of Commonly Used Biomolecules That Differ in Size and Affinity on Tumor Uptake and Internalization
Source: PLoS One. 2015 Apr 22;10(4):e0124440. doi: 10.1371/journal.pone.0124440 (PMC4406587; doi:10.1371/journal.pone.0124440)
Supplement: S1 Table — Two-way ANOVA was used to compare the %ID/ml in the blood of different imaging agents to different time points, followed by Bonferroni post-hoc test. ns = not significant, *p < 0.05, and ***p < 0.001, n = 3 for all except n = 6 for R6.5 and control IgG at 1, 4, 12 and 24 h postinjection. Abbreviations and notations used: mIgG = control IgG; RIgG = R6.5 IgG; BSA = bovine serum albumin; Strep = streptavidin; mFab = control Fab; RFab = R6.5 Fab. (DOCX) [file pone.0124440.s001.docx]

**S1 Table. Two-way ANOVA of the data shown in Figure 2A**

| Time (h) | mIgG vs. | | | | | RIgG vs. | | | | mFab vs. | | | RFab vs. | | BSA vs. |
| --- | --- | --- | --- | --- | --- | --- | --- | --- | --- | --- | --- | --- | --- | --- | --- |
|  | RIgG | mFab | RFab | BSA | Strep | mFab | RFab | BSA | Strep | RFab | BSA | Strep | BSA | Strep | Strep |
| 1 | * | *** | *** | ns | ns | *** | *** | ns | ns | ns | *** | *** | *** | *** | ns |
| 4 | *** | *** | *** | * | ** | *** | *** | *** | *** | ns | *** | *** | *** | *** | ns |
| 8 | * | *** | *** | *** | *** | *** | *** | *** | *** | ns | *** | *** | *** | *** | ns |
| 12 | *** | *** | *** | * | ** | *** | *** | *** | *** | ns | ns | ns | ns | ns | ns |
| 24 | ns | *** | *** | *** | *** | *** | *** | *** | *** | ns | ns | ns | ns | ns | ns |
| Two-way ANOVA was used to compare the %ID/ml in the blood of different imaging agents to different time points, followed by Bonferroni post-hoc test. ns = not significant, *p < 0.05, and ***p < 0.001, n = 3 for all except n = 6 for R6.5 and control IgG at 1, 4, 12 and 24 h postinjection. Abbreviations and notations used: mIgG = control IgG; RIgG = R6.5 IgG; BSA = bovine serum albumin; Strep = streptavidin; mFab = control Fab; RFab = R6.5 Fab | | | | | | | | | | | | | | | |
